# The role of the intestinal microbiome in antiphospholipid syndrome

**DOI:** 10.3389/fimmu.2022.954764

**Published:** 2022-11-24

**Authors:** Dagmar J. M. van Mourik, Dorien M. Salet, Saskia Middeldorp, Max Nieuwdorp, Thijs E. van Mens

**Affiliations:** ^1^ Amsterdam UMC location University of Amsterdam, Department of (Experimental) Vascular Medicine, Amsterdam, Netherlands; ^2^ Amsterdam Cardiovascular Sciences, Pulmonary hypertension & thrombosis, Amsterdam, Netherlands; ^3^ Department of Internal Medicine & Radboud Institute of Health Sciences (RIHS), Radboud University Medical Center, Nijmegen, Netherlands; ^4^ Amsterdam Reproduction & Development, Pregnancy & Birth, Amsterdam, Netherlands

**Keywords:** antiphospholipid syndrome, intestinal microbiome, systemic autoimmune disease, molecular mimicry, diet

## Abstract

The antiphospholipid syndrome (APS) is a thrombotic autoimmune disease in which the origin of the disease-characterizing autoantibodies is unknown. Increased research effort into the role of the intestinal microbiome in autoimmunity has produced new insights in this field. This scoping review focusses on the gut microbiome in its relation to APS. EMBASE and MEDLINE were searched for original studies with relevance to the relation between the gut microbiome and APS. Thirty studies were included. Work on systemic lupus erythematosus, which strongly overlaps with APS, has shown that patients often display an altered gut microbiome composition, that the disease is transferable with the microbiome, and that microbiome manipulation affects disease activity in murine lupus models. The latter has also been shown for APS, although data on microbiome composition is less consistent. APS patients do display an altered intestinal IgA response. Evidence has accrued for molecular mimicry as an explanatory mechanism for these observations in APS and other autoimmune diseases. Specific gut microbes express proteins with homology to immunodominant APS autoantigens. The disease phenotype appears to be dependent on these mimicking proteins in an APS mouse model, and human APS B- and T-cells indeed cross-react with these mimics. Pre-clinical evidence furthermore suggests that diet may influence autoimmunity through the microbiome, as may microbial short chain fatty acid production, though this has not been studied in APS. Lastly, the microbiome has been shown to affect key drivers of thrombosis, and may thus affect APS severity through non-immunological mechanisms. Overall, these observations demonstrate the impact of the intestinal microbiome on autoimmunity and the importance of understanding its role in APS.

## Introduction

Antiphospholipid syndrome (APS) is an autoimmune disease defined by the persistent presence of antiphospholipid antibodies (aPL), as well as clinical complications like thrombotic events and/or obstetric complications. The main autoantigen is β2-Glycoprotein 1 (β2-GP1), formerly known as apolipoprotein H (1). The trigger for autoantibody formation is unknown, as is the exact mechanism of how the antibodies result in thrombus formation. It is believed that a second hit is needed for APS to manifest (2). This second hit can be caused by stress, such as surgery, an infection, or pregnancy. Because the exact etiology of APS is unknown, treatment is based on secondary prevention using anticoagulation instead of curative therapy (3). Identification of etiological factors may help treat this disease more precisely. Recent findings implicate the intestinal microbiome as a causal factor. This scoping review focusses on the developments in the field of the gut microbiome in relation to APS, and also discusses microbiome related observations in other autoimmune diseases where relevant to APS.

## Methods

We performed a scoping review according to the PRISMA guidelines (4). Inclusion criteria were original data studies with relevance to the topic of the review: intestinal microbiome in relation to APS. Studies on other diseases were only included when relevant to this main topic. For a systematic overview of the relation of the microbiome with systemic lupus erythematosus we would like to refer to previously published papers by others (5). Reviews, abstracts and case-reports were excluded, as well as non-English papers. We searched in EMBASE and MEDLINE. Antiphospholipid syndrome, systemic autoimmune disease, intestinal microbe, and gut microbiome were among the used search terms. The complete search strategy and flow diagram are shown in **Appendix 1**. The search resulted in 3750 hits. After removing duplicates, 2965 articles were eligible for title and abstract screening. We selected 167 articles for full text review and 30 studies were included. An overview of the included studies is shown in **Appendix 2**. Screening and full text review was performed by two independent authors. Conflicting evaluations were resolved by a third author.

## The intestinal microbiome and APS

The gut microbiome comprises a diverse metabolically active ecosystem that constantly interacts with the immune system in the intestinal microenvironment. The community consists mainly of a myriad of bacterial strains that are not thought to be pathogenic during homeostasis. Infectious triggers are known to be able to elicit aPL. Examples include human immunodeficiency virus and Sars-CoV2, as well as pathogenic bacteria such as *Mycoplasma pneumonia* and *Streptococcus* spp (35, 36). These infection-triggered aPL are transient, and are likely not thrombogenic (37). The hallmark of APS is the persistent presence of aPL, and the origin of these transient aPL in APS patients is unknown. Exposure to commensal intestinal microbes may act as a chronic autoimmune stimulus that could explain continuous antibody secretion. Mechanisms may involve bacterial translocation, bacterial antigen autoantigen cross-reactivity, bacterial metabolites acting upon the host immune system, and non-immunological APS disease modifiers. These mechanisms are discussed in more detail below.

Few studies have investigated the gut microbial composition in APS. A systemic autoimmune disease cohort comprising of patients with systemic lupus erythematosus (SLE), APS, Sjögren’s syndrome, and undifferentiated connective tissue disease was compared to healthy controls to identify a common gut microbial pattern in these autoimmune diseases (15). The study showed a reduction of tolerogenic microbes in the cohort compared to healthy controls. *Bifidobacterium*, *Streptococcus* and *Ruminiclostridium* bacteria were increased in patients. These bacteria are related to intestinal inflammation and permeability suggesting a role for the microbiome in these autoimmune diseases. SLE and APS patients showed increased relative abundance of *Collinsella*, which was previously found in rheumatic arthritis patients and is correlated with IL-17a production (15, 16). In another study with 22 APS patients and 19 healthy donors, a decrease of *Bilophila* genus and increase in genus *Slackia* in APS patients was observed (6). On the contrary, a third study found no differences in composition between APS patients and controls (27). However, this study also determined gut microbiota IgA coating with fluorescence-activating cell sorting. They found a slight increase of IgA coated bacteria in APS compared to controls. These IgA coated fractions were sequenced, which revealed a different composition of the IgA-coated fraction of the microbiome in APS patients. In addition, A lower α- and β-diversity was observed within the IgA coated fraction of the APS group compared to the controls. Thus, although the overall microbial composition showed no difference between APS patients and controls in this study, APS patients did show a distinct IgA response. IgA coated fractions consist mostly of commensals that are tolerated by the mucosal immune system; a change in the diversity of fractions may reflect a change of tolerance to these different bacteria in mucosal immunity (38). Overall, there is some evidence for a different microbiome composition in APS patients but results are inconsistent.

### Examples from systemic lupus erythematosus with relevance to the microbiome-APS interaction

Work on other autoimmune diseases, especially on SLE, supports a possible role of the microbiome in APS. SLE and APS show strong overlap, with APS patients having an 80 times higher risk of developing SLE (39). Vice versa, 15%-34% of the SLE patients are positive for lupus anticoagulant, 12%-44% have anticardiolipin antibodies and 10%-19% contain anti-β2-GP1 antibodies (40). Approximately 20-50% of these SLE patients with aPLs develop thrombotic events (40). The two syndromes can share clinical features even when they stand alone, such as thrombocytopenia and Libman-Sacks endocarditis, with similar demographics and comorbidities of afflicted individuals.

Many studies examined the microbiome composition in SLE patients. A decrease in microbial diversity has often been observed (8–12). The ratio of *Firmicutes* to *Bacteroidetes* was distinct in SLE patients (13, 14). SLE patients had reduced abundance of *Firmicutes* and *Lactobacillus* (14). This study found that the ratio *Firmicutes* to *Bacteroidetes* was inversely correlated with disease activity represented by the Systemic Lupus Disease Activity Index (r = −0.451; p=0.04) (14). An increase of *Bifidobacteria*, *Proteobacteria* and *Ruminococcus gnavus* was observed in other studies in SLE patients (9, 20). In a cohort of children with SLE, a decrease of *Ruminococcae* and an increase in *Proteobacteria* was found, and both observations were linked to lupus nephritis (11). Overall, most studies reveal a distinct microbiome composition in SLE patients compared to healthy controls. The possibility of publication bias in these types of studies cannot be ruled out. However, experimental work appears to underline these observations in humans.

Several SLE mouse models have been employed to study the role of the microbiome. Experiments using female MRL/*lpr* mice, a genetic SLE model, showed that permeability of the gut was increased before kidney disease development (25). Remarkably, *Lactobacillales* colonization of the intestines reestablished the mucosal barrier function of the gut and decreased the severity of kidney disease. Evidence for a different microbiome composition in lupus mice was demonstrated by a changed *Firmicutes* to *Bacteroidetes* ratio and β-diversity (33). Another study with NZB/W F1 mice showed that upon lupus onset, the gut microbiota is altered (17). These studies show that the microbiome composition in SLE mice is altered compared to healthy mice, which is in line with the human studies, although the direction of causality remains elusive.

Stronger evidence for a causally involved microbe comes from work into the role of *Enterococcus gallinarum* in SLE (22). Gut barrier dysfunction enables *E. gallinarum* to translocate to the liver in some SLE patients. The translocation activates the usually tolerant immune system, which results in an immune response and formation of antibodies against *E. gallinarum*. This autoimmune response is hypothesized to contribute to disease progression in SLE mice. Vaccination with heat-killed *E. gallinarum* reduced serum autoantibodies and prolonged survival of NZB/W F1 mice, while vaccination with other bacteria did not show these improvements. These findings suggest translocation of *E. gallinarum* as a causative agent for the immune response and disease progression. Given the overlap of SLE with APS, these combined results collectively support a possible role for the microbiome in APS.

### Experimental perturbation of the gut microbiome in APS and related diseases

An experimental approach focusing on changing the microbiome composition in APS was studied in several APS- and other autoimmune mouse models. Administration of vancomycin in an APS mouse model, (NZW x BXSB)F1, was shown to reduce anti-β2-GP1 antibody production (22). A study with MRL/*lpr* mice showed that a mix of antibiotics reduced SLE symptoms, and administration of vancomycin led to restoration of the gut barrier (24). The diseased mice showed an increase of *Lachnospiracheae*, which belong to *Clostridia*, and accordingly vancomycin treatment was effective since it is known to deplete *Clostridia* (24). Another study showed similar results for both broad-spectrum antibiotics and vancomycin treatment in SLE mice (22). In accordance with this, a decrease in anti-β2-GP1 titers was observed after treatment with vancomycin or ampicillin, which also prevented mortality (22). The treated mice had lower levels of Th17 and T follicular helper cells in secondary lymphoid tissues and reduced autoantibodies. An increased barrier integrity of the gut was observed as well. In contrast, lupus-prone NZB/W F1 mice were treated with a broad-spectrum antibiotic, which did not affect antibody production and disease symptoms (28). A broad-spectrum antibiotic was also used in a transgenic spontaneous autoimmune myocarditis mouse model and again showed protective effects (18). The antibiotic treatment reduced cardiac inflammation and presence of specific *Bacteroides thetaiotaomicron* IgG antibodies. Both observations associated with improved disease course and reduced risk on mortality. In addition to changing the microbiome composition using antibiotic treatment, administration of gut microbes may also reduce microbiome initiated autoimmune manifestations. Two studies applied *Lactobacilli* as probiotic alone or in combination with tacrolimus, an immunosuppressant used in SLE, as treatment for SLE in mice (31, 41). Both models showed a reduction in symptoms.

Another means of altering the microbiome composition is through a fecal microbiota transplant (FMT). FMT from SLE prone mice into germ-free mice resulted in increased autoantibodies and immune response (21). The FMT also changed immune cell-activation and upregulated expression of lupus susceptibility genes IRF7 and CSK (21). The transferability of the phenotype by the microbiome implicates this as a central factor, at least in this model.

### Molecular mimicry of gut commensals may contribute to autoimmunity

Molecular mimicry is the concept of structural resemblance of a self-peptide of the host and a foreign peptide. Studies regarding molecular mimicry often describe mimicry by pathogenic microbes. There are not many studies describing molecular mimicry in gut commensals. Commensals are regarded to live in symbiosis with the host, and the host immune system tolerates these microbes. However, recent studies showed molecular mimicry of gut commensals promoting autoimmune disease progression. For instance, an extensive translational study on autoimmune myocarditis showed that *B. thetaiotaomicron (B. theta)* expresses autoantigen-mimicking peptides that can activate MYH6-specific CD4+ T-cells (18). These CD4+ T-cells are able to induce cardiomyopathy, an often-lethal disease. In a transgenic MYH6-overexpressing T-cell mouse model, the use of antibiotics led to reduction of cardiac inflammation and reduced the risk of developing cardiomyopathy. Reactivity to *B. theta* was further tested in a human cohort. Sera of patients reacted stronger to *B. theta*-specific IgG compared to sera of healthy controls. Patients with a high response also had a significantly higher disease severity. In a final experiment, mice received an FMT from patients that are either positive or negative for *B. theta*. Mice that received *B. theta* positive FMT suffered from increased cardiac inflammation. These results suggest that *B. theta*, a common gut microbe, could contribute to autoimmunity through molecular mimicry.

Interestingly, this concept has also been shown in APS (27). β2-GP1, an abundantly circulating protein, is targeted by autoantibodies in the majority of patients with APS. The genome of *Roseburia intestinalis*, another common gut commensal, encodes for two mimicking proteins. These contain peptide sequences with high homology to respectively a B-cell and a T-cell auto-epitope in β2-GP1. The *R. intestinalis* protein DNA methyltransferase mimics the immunodominant B-cell epitope, and another protein mimics an important APS T-cell epitope in β2-GP1. The abundance of *R. intestinalis* was not different between APS patients and healthy controls, nor was the IgA coated *R. intestinalis* fraction. However, the APS patients showed strong IgG reactions to the *R. intestinalis* DNA methyltransferase compared to healthy controls, which correlated with the anti-β2-GP1 in the patients. A human derived anti-β2-GP1 monoclonal antibody also showed high binding to the *R. intestinal* mimic, and BALB/c mice immunized with the bacterium developed β2-GP1 reactive antibodies. Similarly, the T-cell mimicking peptide was able to activate APS patient derived T-cells. To verify the causal role in APS pathophysiology, (NZW X BXSB)F1 hybrid male mice, a genetic APS model, were orally administered *R. intestinali*s after pre-treatment with vancomycin. This resulted in development of anti-human β2-GP1 and lethal thrombosis. This work provides convincing evidence for a pathogenic role of a gut commensal in APS through molecular mimicry.

Two additional studies provided evidence of molecular mimicry in autoimmunity in other diseases. Several proteins containing mimotopes were identified that activate T-lymphocytes in uveitis (42). These mimotope-activated T-lymphocytes can cross the blood-retina barrier and recruit inflammatory cells, which cause uveitis (42). Ro60 is an RNA-binding protein and some SLE patients exhibit anti-Ro60 antibodies (19). Ro60 orthologs were found in commensal microbes and it was shown that patients display immune responses to these Ro60 orthologs.

These studies demonstrate that gut commensals are able to mimic molecules of the host, which appears to propagate autoimmune responses and advance autoimmune disease-inflicted end-organ damage, in APS as well as in other diseases.

### Diet and APS

The effect of diet on the composition of microbiome is not fully resolved in the context of autoimmune disease, but several studies did evaluate diet and its effects on the microbial balance and autoimmune disease progression. For instance, Western diet fed autoimmunity-prone mice developed lupus (32). Clinical manifestations in this model were preceded by shifts in the microbiome composition, suggesting a role for the microbiome and perhaps the possibility of early gut microbiome directed intervention. Indeed, dietary modifications in another lupus model protected against disease progression in a microbiome-mediated manner (34). In contrast, a study in humans found no association between a Western diet, considered unhealthy, and the risk of developing SLE or anti-dsDNA antibodies as compared to a prudent diet (30). The Western diet consisted of, for example, white bread, red meat, potatoes, pizza, desserts and sweets, whereas a prudent diet was characterized by whole grains, poultry, fish, legumes, fruit, and vegetables. One theoretical reason that the murine findings did not translate into humans may be an inherently larger inter-individual variability in humans compared to isogenic controlled-diet fed mice. More specifically for APS, the effect of supplementation of a probiotic on acquired immunity was investigated (7). BALB/c mice were immunized with human β2-GP1 and administered *Lactobacillus casei*, commercial yoghurt or PBS daily for two weeks. The mice administered *Lactobacillus casei* displayed a decrease of IL-10 and an increase of IFN-γ secretion by lymphocytes, and a decrease of anti-β2-GP1 antibody titers. Lastly, an attempt to induce oral tolerance through β2-GP1 supplementation is noteworthy in this context. BALB/c mice immunized with human β2-GP1 were administered either purified human β2-GP1, domain I of β2-GP1, domain V of β2-GP1 or PBS weekly (29). A reduction of anti-β2-GP1 antibodies was observed in mice administered either whole β2-GP1 or domain I. Attenuation in thrombus formation and reduction of fetal loss were observed in both groups as well.

### Short chain fatty acids and autoimmunity

Short chain fatty acids (SCFA) are produced by gut microbes and dysbiosis changes the level of SCFA production. SCFAs affect the immune system, for example through stimulation of regulatory T cells (43). In various autoimmune diseases, a role for SCFA has been proposed. Increased serum levels of SCFAs acetate and butyrate protect against diabetes mellitus type 1 in mice (23). In another study, low fecal butyrate was found in patients with rheumatic arthritis and arthritic mice (26). Administration of butyrate to arthritic mice resulted in reduction of disease progression. In one lupus mouse model however, oral SCFA administration did not affect disease activity (28). In APS patients, no data on SCFA levels has been published.

### Non-immunological microbiome related disease modifiers in APS

Non-immunological microbiome-related mechanisms may also contribute to the thrombotic complications of APS, as research has shown effects of the microbiome on the key thrombotic drivers of platelet aggregation and tissue factor expression. Two gut microbiome dependent plasma metabolites, phenylacetylglutamine and trimethylamine N-oxide are independent predictors of cardiovascular events in large population studies (44, 45). Both induced platelet hyper-reactivity and increased thrombotic tendency in mouse models. This aligns with results from supplementation studies that showed effects on platelets in humans (44). Furthermore, the majority of serotonin, a paracrine platelet activator, is produced in the gut by enterochromaffin cells (46). A study using germ free mice showed that gut microbes affect enterochromaffin cells to increase serotonin production, with effects on platelet reactivity and hemostatic tendency upon vascular injury in the animals (46). Other research investigated the mechanism of gut microbiome vascular remodeling in the small intestine using germ-free mice (47). This study showed that the microbiome influences tissue factor glycosylation and cell surface translocation, and thereby downstream thrombin formation (47). These studies show that there are non-immunological microbiome-related mechanisms affecting coagulation.

## Conclusion

The exact etiology of APS is unknown and currently the role of the microbiome in autoimmune diseases is being elucidated. This scoping review discussed the role of the gut microbiome in APS and examples from related autoimmune diseases. Suggestions to guide future research in APS and the intestinal microbiome are presented as an overview of relevant open questions in **Table 1**. The microbiome composition in patients with SLE, a disease that strongly overlaps with APS, is distinct from healthy controls. This was not observed as consistently in APS patients, although an altered intestinal IgA response appears to be present in APS patients. Experimental data show that manipulation of the microbiome in mice affects autoimmune disease activity, including in APS models, supporting a causative role of the microbiome. Molecular mimicry of immunodominant autoantigens by gut microbial proteins may contribute to a sustained autoimmune response in APS, which was also observed in studies on other autoimmune diseases. Diet influences the microbiome composition, the autoimmune phenotype, and disease severity in murine models. This review underlines the complexity of the contribution of the microbiome composition to autoimmunity in APS, although conclusions had to be drawn in part based on related autoimmune disease models given the scarcity of APS microbiome research. Further verification of a causal role of the gut microbiome in APS pathophysiology may facilitate the search for new therapies.

**Table 1:** Open questions in the APS-microbiome field.

Findings relevant for APS	Open questions in APS
Intestinal microbiome composition	• Verification studies with larger cohorts are needed to reveal microbiome composition in APS patients. • What is the role of the IgA coated bacteria in APS?
Experimental perturbation of the gut microbiome	• Can antibiotic or other microbial treatment reduce disease activity in human APS?
Molecular mimicry	• Is molecular mimicry present in all APS patients? • Does molecular mimicry contribute to disease in human APS? • What specific commensals show molecular mimicry in APS patients?
Diet and short chain fatty acids	• Can diet inhibit the development or influence disease activity of APS? • Do short chain fatty acids affect APS?

## Data availability statement

The original contributions presented in the study are included in the article/Supplementary Material. Further inquiries can be directed to the corresponding authors.

## Author contributions

DM wrote the manuscript. DM and DS performed the literature search and screening. TM resolved conflicts from the screening. DM, DS, and TM revised the manuscript. All authors contributed to the article and approved the submitted version.

## Conflict of interest

SM reports grants from GSK, Aspen, and Pfizer, grants and personal fees from Daiichi-Sankyo, Bayer, Pfizer, and Boehringer-Ingelheim, personal fees from Portola/Alexion, Abbvie, Pfizer/Bristol-Meyers Squibb, Norgine, Viatris, and Sanofi, outside the submitted work. MN is part of the Scientific Advisory Board of Caelus Health, The Netherlands.

The remaining authors declare that the research was conducted in the absence of any commercial or financial relationships that could be construed as a potential conflict of interest.

## Publisher’s note

All claims expressed in this article are solely those of the authors and do not necessarily represent those of their affiliated organizations, or those of the publisher, the editors and the reviewers. Any product that may be evaluated in this article, or claim that may be made by its manufacturer, is not guaranteed or endorsed by the publisher.

## Figures and Tables

**Figure:**
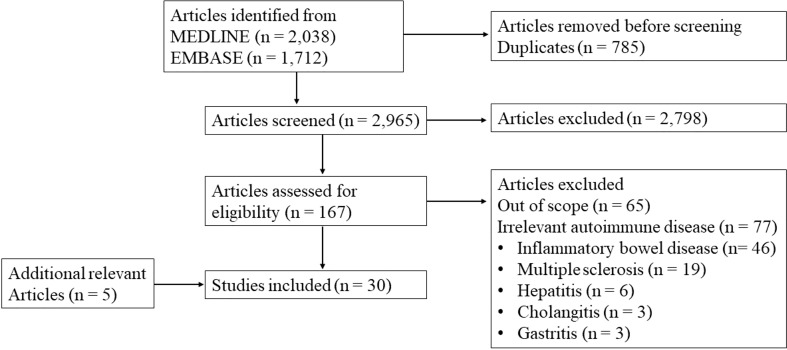
Flow diagram of the screening.

## Appendix 1 Search strategy and flow diagram

**Appendix Table 1:** Search strategy in EMBASE.

#	EMBASE search performed at 23.03.2022	Results
1	‘antiphospholipid syndrome’/exp OR ‘antiphospholipid syndrome’	20248
2	‘systemic autoimmune disease’/exp OR ‘systemic autoimmune disease’	3254
3	chronic AND autoimmune AND (‘disease’/exp OR disease)	62841
4	‘phospholipid antibody’/exp OR ‘phospholipid antibody’	15180
5	#1 OR #2 OR #3 OR #4	92561
6	‘intestine flora’/exp OR ‘intestine flora’	76811
7	(‘gut’/exp OR gut) AND microb*	110880
8	intestinal AND microb*	72152
9	gastrointestinal AND microb*	56448
10	gastric AND microb*	17813
11	(‘intestine’/exp OR intestine) AND (‘microflora’/exp OR microflora)	84863
12	(‘gut’/exp OR gut) AND (‘microflora’/exp OR microflora)	68802
13	microb*:ab,ti AND (‘gut’/exp OR gut OR intestin* OR gastrointestin*)	103174
14	‘Molecular mimicry’	8516
15	#6 OR #7 OR #8 OR #9 OR #10 OR #11 OR #12 OR #13 OR #14	208359
16	#5 AND #14	1712

**Appendix Table 2:** Search strategy in MEDLINE.

#	MEDLINE search performed at 23.03.2022	Results
1	(Antiphospholipid syndrome) OR (Anti-phospholipid syndrome) OR (antiphospholipid syndrome[Mesh terms]) OR (Systemic autoimmune disease) OR (Systemic autoimmune disorder) OR (Chronic autoimmune disease) OR (Chronic autoimmune disorder) OR (Antibodies, antiphospholipid[Mesh terms])	167789
2	(gastrointestinal microbiome[Mesh terms]) OR (gut microb*) OR (intestinal microb*) OR (gastrointestinal microb*) OR (gastric microb*) OR (intestine flora) OR (intestine microflora) OR (gut microflora) OR (molecular mimicry) OR (microb*[tiab] AND (gut OR intestin* OR gastrointestin*))	225805
3	#1 AND #2	2038

## Appendix 2 Overview included studies

**Appendix Table 3:** Overview of the included studies.

Author, year	Type of study	Summary of the findings	Reference
Aguiar et al., 2016 Arthritis and Rheumatology	Human cohort	Intestinal microbiome composition is distinct in APS patients compared to healthy controls.	(6)
Amital et al., 2007 Ann N Y Acad Sci.	Animal study	Probiotic supplementation in an APS mouse model resulted in reduced anti-β2-GP1 titers.	(7)
Azzouz et al., 2019 Ann Rheum Dis. Chen et al., 2021 Arthritis Rheumatol. Gerges et al., 2021 Int J Microbiol. Hevia et al., 2014 mBio. Li et al., 2019 Clin Sci (Lond). Liu et al., 2021 Front Immunol. Wen et al., 2021 J Immunol Res.	Human cohorts	The intestinal microbiome composition is altered in SLE patients compared to healthy controls.	(8–14)
Bellocchi et al., 2019 J Clin Med.	Human cohort	Amount of tolerogenic microbes is reduced in autoimmune disease-cohort compared to healthy controls.	(15)
Chen et al., 2016 Genome Med.	Translational	Rheumatoid arthritis patients have decreased intestinal microbiome diversity, which correlated with antibody levels.	(16)
Chen et al., 2021 Sci Rep.	Animal study	Upon SLE onset, the microbiome is changed in SLE-prone mice.	(17)
Gil-Cruz et al., 2019 Science.	Translational	Evidence for causal T-cell cross-reactivity between gut microbial proteins and autoantigens in autoimmune myocarditis.	(18)
Greiling et al., 2018 Science Translational Medicine.	Translational	Evidence for causal cross-reactivity between gut microbial proteins and autoantigens in SLE.	(19)
Kim et al., 2021 Front Immunol.	Animal study	Treatment with probiotic showed a reduction of lupus symptoms and increased efficacy of tacrolimus.	(41)
Luo et al., 2018 Appl Environ Microbiol.	Translational	In lupus patients, an increase of *Bifidobacteria, Proteobacteria* and *Ruminococcus gnavus* was observed.	(20)
Ma et al., 2019 Molecular Medicine.	Animal study	Fecal microbiota transplantation of lupus-mice led to increased autoantibody formation and immune response in germ-free mice.	(21)
Manfredo Vieira et al., 2018 Science.	Translational	Translocation of a gut microbe, *Enterococcus gallinarum*, induces autoimmune responses and progression of SLE symptoms.	(22)
Marino et al., 2017 Nat Immunol.	Animal study	Increased serum levels of some short chain fatty acids are protective against diabetes mellitus type 1 in mice.	(23)
Mu et al., 2017 Sci Rep.	Animal study	Administration of antibiotics diminished SLE symptoms and restored the intestinal barrier.	(24)
Mu et al., 2017 Microbiome.	Animal study	Gut permeability precedes kidney disease in a genetic SLE mouse model. This was reversed by colonization of *Lactobacillalus*.	(25)
Rosser et al., 2020 Cell Metab.	Translational	In patients and mice with rheumatic arthritis low fecal butyrate was observed.	(26)
Ruff et al., 2019 Cell Host Microbe.	Translational	Evidence for causal cross-reactivity between gut commensal *Roseburia intestinalis* proteins and autoantigens in APS.	(27)
Schäfer et al., 2021 Front Immunol.	Animal study	Treatment with broad-spectrum antibiotics were ineffective against antibody production or SLE symptoms.	(28)
Shemer et al., 2019 J Autoimmun.	Animal study	In an APS murine model, oral administration of β2-GP1 resulted in reduced anti-β2-GP1 titers and diminished thrombus formation.	(29)
Tedeschi et al., 2020 Lupus.	Human cohort	No relation between diet and SLE symptoms were identified.	(30)
Toral et al., 2019 Faseb j.	Animal study	Treatment with probiotic showed a reduction of lupus symptoms.	(31)
Vorobyev et al., 2019 Nat Commun.	Animal study	Lupus developed, preceded by microbiome changes, in autoimmunity-prone mice fed with a Western diet.	(32)
Wang et al., 2021 Front Immunol.	Animal study	Lupus mice contain a distinct microbiome composition.	(33)
Zegarra-Ruiz et al., 2019 Cell Host Microbe.	Animal study	Dietary modifications in a lupus model protected against disease progression in a microbiome-mediated manner.	(34)

β2-GP1 – β2-Glycoprotein 1, APS – antiphospholipid syndrome, SLE – systemic lupus erythematosus.
